# Long-Acting Reversible Contraceptive Use by Rural–Urban Residence among Women in Nigeria, 2016–2018

**DOI:** 10.3390/ijerph192013027

**Published:** 2022-10-11

**Authors:** Otobo I. Ujah, Russell S. Kirby

**Affiliations:** College of Public Health, University of South Florida, Tampa, FL 33620, USA

**Keywords:** long-acting reversible contraception, rural, urban, IUD, implant, PMA, Nigeria

## Abstract

This study examined temporal trends in the association between rural–urban residence and the use of LARCs among women using a method of contraception. A secondary objective was to examine whether the association varied over time. This study was a secondary analysis of data collected by the Performance Monitoring for Action (PMA) project from Nigeria among women aged 15–49 in 2016 (*N*= 11,054), 2017 (*N*= 11,380), and 2018 (*N* = 11,106). Weighted multivariable logistic regression analyses examined the association between place of residence and the likelihood of LARC (overall and specific type) utilization. Using weighted multivariable logistic regression, we show that, of the 6488 women who were using a method of contraception, the rates of LARC utilization in urban areas were significantly lower than in rural areas (OR = 0.52, 95% CI 0.38–0.73), attributed mainly to the high utilization rates of implants. Women in urban areas were more likely to use intrauterine devices (IUDs) (OR = 1.90, 95% CI 1.09–3.30) compared to those in rural areas. Conversely, the use of implants was significantly lower among women in urban areas (OR = 0.39, 95% CI 0.28–0.54). Adjusting for all predictors, we observed a reduction, albeit not significantly different, in odds in overall LARC, IUD, and implant use in urban compared to rural areas. The use of LARCs increased between 2016 and 2018 and the association between LARC use and place of residence also differed by the PMA survey year. There is a need for programs and policies to close gaps in the disparities in overall and specific LARC utilization rates based on place of residence.

## 1. Introduction

As the rate of population growth in Sub-Saharan Africa (SSA) continues to rise, there are concerns about the detrimental effect this poses to socioeconomic development within the region [1]. Expanding access to modern contraceptive methods has been advocated as an effective strategy to slow down population growth, yet not many women who desire to space or limit their births use a method of contraception, hence the high unmet need for contraception [1,2,3]. In Africa, the modern contraceptive prevalence rate (mCPR) varies by geographical location, being as high as 62% in Southern Africa to as low as 9% in Senegal. In Nigeria, the mCPR has been estimated to be about 12% [1,2]. The use of contraception is associated with significant declines in the rates of unintended pregnancies and the attendant sequelae of unsafe abortions, as well as the unacceptably high maternal morbidity and mortality rates [1].

Modern contraceptive methods are classified into short-acting and long-acting (reversible or permanent) methods. The long-acting reversible contraceptives (LARCs) comprise subdermal implants, a copper-bearing intrauterine device (Cu-IUD), and the levonorgestrel-releasing intrauterine system (LNG-IUS) [4,5]. These methods are deemed efficient methods of contraception given that they are durable, cost-effective, and associated with low failure rates [1,4,6]. Unfortunately, LARC uptake and utilization in Sub-Saharan African populations have been suboptimal, attributable to barriers on both the supply and demand sides [7]. 

While studies have examined trends in access to and utilization of contraceptives in Sub-Saharan Africa, research on LARCs remains scarce, and only a few studies have focused on LARC uptake in Nigeria [1,5,8,9,10]. Examining trends in differential uptake of LARCs between women residing in rural and urban settings in Nigeria will enhance understanding of determinants of uptake in these contexts, as well as tailor interventions that will drive equity in contraceptive availability, accessibility, and uptake. 

Therefore, the objective of this study was to examine temporal trends in the association between rural–urban residence and the use of LARCs among women using a method of contraception, and secondarily, examine whether the association varied over time.

## 2. Methods

This study was based on a secondary analysis of publicly available data from population-based surveys conducted by Performance Monitoring for Action (PMA) 2020 in Nigeria. The PMA2020 survey uses a stratified multi-stage cluster sampling design to obtain a probability sample of (sub)nationally representative households and females 15–49 years [11,12]. Data are collected in 11 countries in Africa and Asia using female resident enumerators (REs) recruited from the sample enumeration areas (EAs) to collect data using smart devices. These surveys are performed yearly, and data collected are made publicly available within 6 months after completion of the surveys. Further details of the survey methodology are contained in Zimmerman et al. [12], and datasets can be obtained from the PMA website at www.pmadata.org (Accessed 15 September 2021).

In this study, nationally representative data from 2016, 2017, and 2018 rounds of the PMA2020 surveys in Nigeria were pooled and used for the analysis [13,14,15]. For the survey, one state was selected per geopolitical zone (*n* = 6) using probability proportional to size. An additional state (Kaduna) was included, and corresponding adjustments were made to the zonal weights resulting in a total of 7 states [11]. A total of 302 clusters of enumeration areas were obtained using the National Population Commission’s (NPC) Census master sampling frame of 2006. Of the total 33,540 women (Round3/2016, 11,054; Round4/2017, 11,380; and Round5/2018, 11,106) sampled during the survey, only approximately 20% (*n* = 6488) reported currently using a contraceptive method.

The samples were adjusted for weights to make them nationally representative. Ethical approval for conducting the PMA2020 survey was obtained from the National Health Research Ethics Committee (NHREC), Department of Health Planning, Research and Statistics; Federal Ministry of Health; and the Johns Hopkins Bloomberg School of Public Health. In addition, informed consent from each respondent was obtained prior to enrollment in the study. 

### 2.1. Measures

The dependent variable for this study was current LARC use (yes vs. no) among women of reproductive age who reported using a method of contraception at the time of the survey. LARC was defined as either implants or intrauterine devices at the time of the study. In addition, we explored the use of implants and IUDs individually relative to other contraceptive methods. The PMA2020 surveys measured current contraceptive use using a series of questions. Initially, respondents were asked, “Are you or your partner currently doing something or using any method to delay or avoid getting pregnant?”. For women who reported using contraception, they were asked to name the method(s) they were using ranging from modern to traditional methods of contraception.

The main independent variable was the place of residence of women. This was defined based on the location of the household as was dichotomized into urban and rural residence. The covariates included were demographic characteristics (maternal age, marital status, family type, place of residence, geopolitical zone, survey year), socioeconomic characteristics (wealth quintile, level of education), reproductive characteristics (number of births, intent for future children), and contraceptive characteristics (knowledge of LARCs, the contraceptive decision-maker at the facility). 

### 2.2. Statistical Analysis

Statistical analyses were performed using the survey analysis procedures in SAS version 9.4 (SAS Institute, Cary, NC, USA) and were restricted to current users of contraception at the time of the survey. The analysis was run on a subsample of women who reported using a method of contraception at the time of the survey (*n* = 6488). The initial analysis involved a weighted univariate analysis to determine differences in the distribution of demographic, socioeconomic, reproductive, and contraceptive characteristics by PMA2020 survey year and place of residence. The overall rates of LARC use, IUD use, and implant use per 100 women were calculated for current users of contraception in rural and urban areas. Thereafter, unadjusted logistic regression analyses of the independent variable and each covariate with current LARC use were performed.

Multivariable (weighted) logistic regressions were then performed to examine associations between place of residence and LARC use, IUD use, and implant use, while controlling for potential confounders which were all identified a priori. In the adjusted models for LARC use, IUD use, and implant use *(n* = 6), Model 1 included sociodemographic characteristics, while in Model 2 contraceptive and reproductive characteristics were added to the model. For all models in this study, the model fit was compared using the Akaike’s Information Criterion (AIC) and the models with smallest AIC were selected as the best-fitting models.

In the final part of the analysis, interaction effects were examined to what extent the association between place of residence and LARC (overall and specific) use varied with the PMA2020 survey year. The regression results are presented as unadjusted and adjusted odds ratios (OR and aOR, respectively) and 95% confidence intervals (95% CI). There were a few missing values that were considered to be missing at random. 

## 3. Results

Overall, among the total population of non-pregnant women aged 15–49 who participated in the PMA survey (*N* = 33,540), 11.7% (*n* = 3917) of women residing in urban areas were using a method of contraception compared to 7.7% (*n* = 2571) of women living in rural areas. Descriptive statistics of the study population characteristics are presented in Table 1. The majority of women resided in urban areas and had a high socioeconomic status. The mean age of the study population was 31.8 ± 0.2 years. Overall, 14.2% of women using a method of contraception were using LARCs, with the majority using implants. In rural areas, more women used implants, while more women in urban areas were using IUDs.

**Figure 1:**
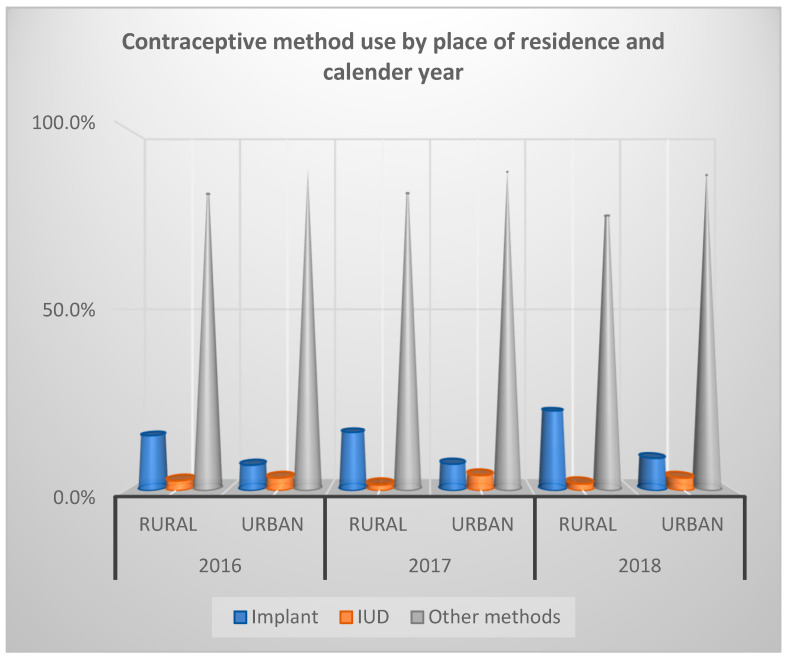
Overall contraceptive utilization rate by place of residence and year of PMA survey among women 15–49 years in Nigeria.

Figure 1 illustrates contraceptive use rate by place of residence and the PMA survey year. We observed that in 2016, 15.1% of women 15–49 years old living in rural areas were using implants, 2.7% were using IUDs, while 82.3% were using other contraceptive methods. Among women living in urban areas, 7.0% were using implants, 3.5% were using IUDs, and 89.5% were using other contraceptive methods. In 2017, of the women 15–49 years old residing in rural areas using contraception, 16.0% were using implants, 1.6% were using IUDs, while 82.5% were using other methods. For women in urban areas, 7.3% were using implants, 4.2 were using IUDs, while 88.5% were using other methods. In 2018, 21.9% of women living in rural areas were using implants, 1.9% were using IUDs, and 76.2% were using other contraceptive methods, while 8.9%, 3.5%, and 87.6% of women living in urban areas were using implants, IUDs, and other contraceptive methods, respectively.

**Figure 2:**
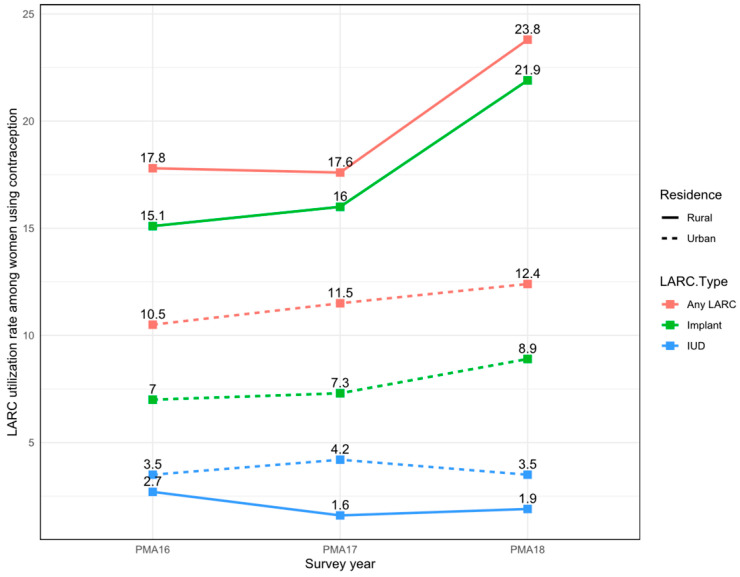
Trends in long-acting reversible contraception (LARC) use, intrauterine device (IUD) use, and implant use PMA2020, 2016–2018. Error bars represent 95% confidence intervals.

The trends in rates of overall LARC use, IUD use, and implant use in rural and urban areas are shown in Figure 2 and Table 2. 

Table 3 shows the unadjusted odds and adjusted odds (Models 1 and 2) from logistic regression models predicting current LARC use. The odds of LARC use were significantly lower among women in urban areas compared with women in rural settings (OR 0.52, 95% CI 0.38–0.73). Although the odds ratio remained lower in both models that adjusted for sociodemographic characteristics (Table 3, Model 1) and in the final model (Table 3, Model 2), these were no longer statistically significant.

Women residing in urban areas had 1.90 times significantly higher odds of using IUDs compared to women residing in rural areas (OR 1.90, 95% CI 1.09–3.30) (Table 4). In the adjusted models (Table 4**,** Models 1 and 2), however, the odds of IUDs were lower, albeit not significantly, among women in urban areas compared to women in rural areas (Model 1: aOR 0.91, 95% CI 0.54–1.54; Model 2: aOR 0.95, 0.57–1.56). Conversely, women residing in urban areas had 61% significantly lower odds of using implants compared to women in rural areas (OR 0.39, 95% CI 0.28–0.54) (Table 5), with the odds remaining lower after adjusting for other covariates (Table 5, Model 1: aOR 0.83, 95% CI 0.59–1.17; aOR 0.79, 0.56–1.11).

**Figure 3:**
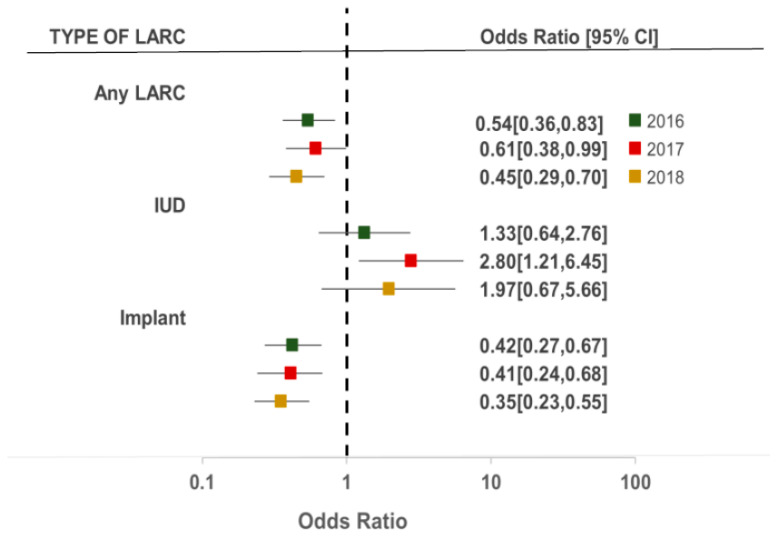
Unadjusted odds ratios of the effect of survey year on the relationship between rural–urban residence and LARC use.

The PMA2020 survey rounds (2016, 2017, and 2018) modified the association between place of residence and LARC use, IUD use, and implant use (Figure 3). For women who reported using a form of LARC, those living in urban areas compared to rural areas had 0.54 times, 0.61 times, and 0.45 times lower odds of using LARCs in 2016, 2017, and 2018, respectively (95% CI 0.36–0.83; 95% CI 0.38–0.99; 95% CI 0.29–0.70). Although the odds of IUD use among women in urban areas was higher compared to those in rural areas in all rounds of the survey (2016, 2017, and 2018), this was only statistically significant in 2017 (OR 2.80, 95% CI 1.21–6.45). The direction of the effect among implant users mirrors patterns observed among those using a form of LARC, with the odds being significantly lower across all years of the survey among women in urban areas compared to those residing in rural areas (2016: OR 0.42, 95% CI 0.27–0.67; 2017: OR 0.41, 95% 0.24–0.68; 2018: OR 0.35, 95% CI 0.23–0.55).

## 4. Discussion

This study showed that place of residence on LARC utilization was associated with the type of LARC being used. Women in rural areas were more likely to use LARCs, a finding which, based on our analysis, was driven largely by the use of implants, while women in urban areas were more likely to use IUDs. The relationship between the use of LARCs regardless of the type was strongest among women who had heard about LARCs. Furthermore, the likelihood of using LARCs based on place of residence varied by year. We found that women in urban areas had an increased likelihood of using IUDs in 2017, while women in rural areas had an increased likelihood of using implants in 2018. These findings suggest that women in rural areas were more likely to use LARCs compared to women in urban areas, with implants being the most commonly used form of LARC in these areas. 

There is a complex relationship between fertility (rate) and contraceptive behavior based on place of residence in Nigeria. While the total fertility rate (TFR) in rural areas is estimated to be about 1.3 times compared to urban areas, the modern contraceptive use, on the other hand, is higher in urban areas compared to rural areas (18.2% vs. 7.8%, respectively) [16,17], thus highlighting a higher unmet need for modern contraception for women in rural areas. With task shifting in contraceptive service provision for women in resource-constrained settings evolving, it is expected that many lower cadre staff will be trained and deployed to rural areas to scale LARC provision [16,18,19]. This likely explains the increased uptake of LARCs among women in rural areas in our study. Marie Stopes Nigeria (MSION) and the International Contraceptive Access (ICA) Foundation have been involved in providing support and training to private sector healthcare providers in Nigeria on LARCs [7,17,20,21,22]. 

The rate of IUD use in our study in 2016, 2017, and 2018 among women residing in both urban and rural areas was consistently less than 5%. This finding is consistent with the evidence in the literature, highlighting the low rate of IUD use of less than 5% compared to other modern contraceptive methods used in many countries, especially in Sub-Saharan Africa [20]. This may be attributable to limited access, myths about IUDs, and medical mistrust [23].

In our study, there was a remarkable increase in the rate of implant use in rural areas between 2017 and 2018, while the rate of IUD remained relatively unchanged during this period in the same setting. This finding is explained by a study in Nigeria [22] which showed that 65% of the 558,571 LARCs provided by five selected program initiatives in Nigeria were implants. While this finding is reassuring from public health and demographic standpoints, it raises concerns about inappropriate LARC-related coercion among poor women given that fertility, contraception, and sexual intercourse are private issues to be determined by individuals [24]. In our study, we found that the odds of using LARCs were significantly higher when the final decision on the contraceptive method to use was made by the provider alone or together with the woman. According to the self-determination theory, autonomously motivated women should make contraceptive decisions that reflect themselves and on their own volition [25].

This study provides evidence not only on rural–urban disparities in LARC use in Nigeria but also regarding disparities in LARCs and short-acting reversible contraceptives (SARCs) using nationally representative data. This study was limited by the fact that ascertainment of the outcome(s) of interest was based on self-report. This could result in misclassification of the outcome and ultimately bias the effect measures. Furthermore, the analysis on LARC use included both modern and traditional contraceptives. This has implications for interpreting modern contraceptive prevalence rates (mCPR), which should be approached with caution.

In conclusion, this present study demonstrates that women residing in rural areas have higher odds of overall LARC use compared to women in urban areas, a finding which was driven by the pattern of implant use in rural areas contrary to women residing in urban areas who had higher odds of using IUDs compared to women in rural areas. It also supports existing evidence of low rates of LARC use in Sub-Saharan Africa. Future research should examine contextual influences among a subpopulation of LARC users, especially among adolescents and young adults, to provide a framework for which interventions can be modified to improve LARC uptake.

## Figures and Tables

**Table 1:** Characteristics of respondents interviewed by rural–urban residence PMA2020, 2016–2018 (*n* = 6488).

	2016		2017		2018	
	Rural (*N* = 778)	Urban (*N* = 1114)	Rural (*N* = 836)	Urban (*N*= 1316)	Rural (*N*= 930)	Urban (*N* = 1435)
**Age, y, mean (SE)**	31.1 (0.41)	31.6 (0.34)	31.8 (0.37)	31.6 (0.36)	31.7 (0.68)	32.2 (0.37)
	*N* (%)	*N* (%)	*N* (%)	*N* (%)	*N* (%)	*N* (%)
**Education** None Primary Secondary Higher	121 (14.4) 180 (21.1) 400 (50.4) 77 (14.1)	51 (2.0) 145 (10.9) 541 (50.9) 376 (36.2)	170 (17.0) 220 (26.4) 347 (40.0) 99 (16.7)	63 (2.4) 150 (10.2) 691 (53.6) 412 (33.8)	160 (16.5) 194 (20.7) 446 (47.8) 130 (15.1)	52 (2.5) 125 (8.0) 741 (51.0) 517 (38.4)
**Marital Status** Married/living together Divorced/separated Never married	620 (75.70 31 (3.3) 127 (21.0)	847 (73.7) 31 (3.4) 236 (23.2)	684 (80.6) 20 (2.6) 132 (16.8)	996 (72.5) 34 (1.8) 286 (25.7)	735 (77.5) 29 (3.3) 166 (19.2)	1060 (72.3) 26 (1.8) 349 (25.9)
**Number of births** 0–1 2–4 5+	181 (27.9) 334 (38.4) 263 (33.7)	342 (33.6) 514 (46.5) 258 (19.9)	61 (8.2) 342 (47.0) 315 (44.8)	139 (13.7) 600 (59.3) 324 (27.0)	235 (26.4) 370 (39.9) 324 (33.7)	480 (35.4) 682 (48.2) 271 (16.4)
**Family type** Monogamous Polygamous	450 (75.2) 166 (24.5)	679 (86.2) 149 (12.3)	458 (71.2) 216 (28.6)	825 (86.8) 167 (12.6)	524 (74.0) 205 (24.8)	887 (86.9) 167 (12.8)
**Heard of LARC** No Yes	192 (24.9) 586 (75.1)	231 (23.5) 883 (76.5)	162 (19.7) 674 (80.3)	182 (17.8) 1134 (82.2)	115 (13.3) 815 (86.7)	170 (13.6) 1265 (86.4)
**Wealth Quintile** 1 2 3 4 5	255 (29.5) 295 (33.2) 113 (12.3) 78 (13.4) 37 (11.6)	14 (0.5) 110 (8.0) 290 (23.9) 341 (30.1) 359 (37.5)	330 (35.8) 261 (26.8) 115 (14.0) 79 (11.5) 51 (11.90	11 (0.5) 188 (13.2) 307 (22.8) 392 (29.7) 418 (33.8)	332 (38.0) 320 (30.8) 135 (13.6) 97 (11.2) 46 (6.4)	18 (0.6) 159 (9.5) 317 (19.7) 453 (33.0) 488 (37.1)
**More children desired** No Yes Cannot get pregnant Undecided/do not know	266 (34.4) 409 (53.0) 18 (3.6) 85 (9.1)	388 (36.1) 634 (55.7) 8 (0.7) 82 (7.5)	297 (36.3) 426 (49.4) 26 (4.3) 84 (9.7)	462 (33.9) 721 (55.6) 16 (0.9) 117(9.7)	342 (38.6) 505 (50.9) 15 (3.5) 67 (7.0)	485 (33.6) 823 (58.1) 14 (0.6) 113 (7.7)
**FP choice decider at facility** Respondent alone Provider Partner Respondent and provider Respondent and partner Other	215 (24.3) 24 (2.4) 67 (9.5) 39 (4.0) 420 (58.6) 7 (1.2)	356 (31.1) 27 (1.5) 100 (9.6) 54 (4.9) 558 (52.2) 9(0.7)	182 (24.3) 27 (3.7) 71 (11.7) 59 (8.4) 326 (51.5) 1 (0.3)	312 (33.6) 43 (2.7) 81 (10.8) 91 (7.8) 393 (44.5) 4 (0.5)	252 (30.5) 32 (8.3) 79 (9.9) 79 (9.7) 307 (41.0) 4 (0.5)	360 (37.2) 46 (3.1) 135 (16.6) 88 (8.1) 370 (34.7) 5 (0.3)
**Contraceptive method** Implant IUD Other methods	120 (15.1) 27 (2.7) 641 (82.3)	108 (7.0) 37 (3.5) 969 (89.5)	161 (16.0) 17 (1.6) 658 (82.5)	156 (7.3) 50 (4.2) 1110 (88.5)	216 (21.9) 13 (1.8) 701 (76.2)	174 (8.9) 54 (3.5) 1207 (87.6)
**State** Kaduna Lagos Taraba Kano River Nasarawa Anambra	233 (16.0) - 69 (16.4) 17 (2.5) 133 (20.6) 225 (33.2) 101 (11.3)	178 (3.2) 371 (41.6) 28 (4.1) 77 (4.4) 210 (24.7) 49 (3.3) 201 (18.7)	264 (15.6) - 65 (16.8) 22 (3.3) 128 (19.1) 254 (34.3) 103 (10.9)	242 (4.5) 456 (41.0) 37 (4.9) 91 (4.3) 215 (23.7) 63 (3.6) 212 (18.0)	282 (11.3) - 88 (19.4) 21 (2.6) 173 (23.1) 229 (32.6) 137 (11.0)	237 (5.0) 511 (42.8) 27 (3.3) 95 (4.5) 235 (22.9) 42 (2.1) 288 (19.4)

Note: Percentages may not sum to 100% due to missingness. Frequencies are raw and percentages are weighted to reflect the Nigerian female population. FP = family planning; IUD = intrauterine device; LARC = long-acting reversible contraceptive.

**Table 2:** Association between year change and rates of LARC use by place of residence.

Place of Residence	Type of LARC	*X* ^2^	*p*-Value ^†^
**Rural**			
	IUD	2.67	0.58
	Implant	16.86	0.035 *
	Any LARC	12.68	0.018 *
**Urban**			
	IUD	1.29	0.66
	Implant	3.78	0.26
	Any LARC	2.24	0.47

Note: ^†^ *p* values are from chi-squared tests computed from the weighted data and set at significance level α = 0.05; LARC = acronym for long-acting reversible contraceptive; IUD = intrauterine device. * Values are statistically significant at α = 0.05.

**Table 3:** Weighted crude and adjusted odds of long-acting reversible contraceptive use among Nigerian women aged 15–49 using contraception aged 15–49.

*N* = 6488	Unadjusted Odds		Adjusted Odds			
	OR	95% CI	Model 1: aOR	95% CI	Model 2: aOR	95% CI
**Place of Residence**						
Rural (ref)	1.00	1.00	1.00	1.00	1.00	1.00
Urban	0.52	0.38–0.73	0.82	0.61–1.12	0.77	0.56–1.06
**Year**						
2016 (ref)	1.00	1.00	1.00	1.00	1.00	1.00
2017	1.04	0.81–1.32	1.06	0.82–1.37	1.45	1.09–1.93
2018	1.29	1.01–1.63	1.46	1.13–1.90	1.91	1.45–2.51
**Age (y)**						
15–24 (ref)	1.00	1.00	1.00	1.00	1.00	1.00
25–34	4.41	3.33–6.01	2.62	1.82–3.77	1.88	1.26–2.81
35–49	5.35	3.68–7.77	2.77	1.87–4.11	1.36	0.84–2.21
**Education**						
None (ref)	1.00	1.00	1.00	1.00	1.00	1.00
Primary	0.72	0.53–0.99	1.09	0.80–1.48	0.98	0.69–1.40
Secondary	0.45	0.32–0.64	1.34	0.96–1.86	1.39	0.94–2.07
Higher	0.51	0.36–0.71	1.55	1.07–2.25	1.70	1.10–2.63
**Marital Status**						
Married/living together (ref)	1.00	1.00	1.00	1.00	1.00	1.00
Divorced/separated	0.83	0.51–1.36	0.73	0.08–6.75	0.55	0.07–4.68
Never married	0.08	0.05–0.14	1.61	0.31–8.42	1.56	0.33–7.40
**Wealth Quintile**						
1 (lowest, ref)	1.00	1.00	1.00	1.00	1.00	1.00
2	0.98	0.62–1.56	1.29	0.93–1.79	1.30	0.92–1.82
3	0.64	0.37–1.12	1.6	1.05–2.44	1.59	0.99–2.55
4	0.55	0.34–0.89	1.55	1.03-2.33	1.53	0.99-2.37
5	0.68	0.43-1.10	2.18	1.37-3.49	2.33	1.40–3.88
**Family type**						
Monogamous (ref)	1.00	1.00	1.00	1.00	1.00	1.00
Polygamous	1.29	1.06–1.58	0.86	0.69–1.07	0.74	0.57–0.94
**State**						
Kaduna	1.00	1.00	1.00	1.00	1.00	1.00
Lagos	0.26	0.19–0.37	0.18	0.12–0.26	0.24	0.16–0.36
Taraba	0.21	0.10–0.47	0.31	0.16–0.62	0.44	0.23–0.83
Kano	0.62	0.39-1.00	0.48	0.32–0.73	0.51	0.29–0.90
River	0.18	0.12–0.26	0.13	0.09–0.20	0.21	0.13–0.34
Nasarawa	0.94	0.67–1.34	1.05	0.72–1.52	1.15	0.77–1.71
Anambra	0.18	0.12–0.27	0.13	0.08–6.75	0.24	0.15–0.38
**Final FP decider at facility**						
Respondent alone (ref)	1.00	1.00			1.00	1.00
Provider	2.51	1.19–5.29			1.32	0.65–2.68
Partner	0.31	0.18–0.54			0.35	0.20–0.63
Respondent and provider	2.51	1.84–3.43			1.48	1.05–2.08
Respondent and partner	0.87	0.68–1.10			0.67	0.53–0.86
Other	0.1	0.01–0.75			0.07	0.01–0.63
**Number of births**						
0–1 (ref)	1.00	1.00			1.00	1.00
2–4	6.7	3.98–8.01			1.92	1.24–2.96
5+	7.34	4.98–10.81			2.32	1.40–3.82
**More children desired**						
No (ref)	1.00	1.00			1.00	1.00
Yes	0.38	0.30–0.47			0.55	0.41–0.74
Cannot get pregnant	0.3	0.09–0.95			0.27	0.10–0.68
Undecided/do not know	0.72	0.51–1.00			0.77	0.53–1.10
**Heard of LARCs**						
No (ref)	1.00	1.00			1.0	1.0
Yes	21.14	10.02–44.57			8.88	4.31–18.28

Note: OR = odds ratio; CI = confidence interval; LARCs = long-acting reversible contraceptives. Results are weighted to reflect the Nigeria female population. The first column shows the unadjusted odds ratio. In the adjusted models, Model 1: sociodemographic characteristics (maternal age, marital status, family type, place of residence, geopolitical zone, survey year wealth quintile, level of education); Model 2 added reproductive characteristics (number of births, intent for future children) and contraceptive characteristics (knowledge of LARCs, the contraceptive decision-maker at the facility).

**Table 4:** Weighted crude and adjusted odds of **intrauterine device (IUD)** use among Nigerian women aged 15–49 using contraception.

*N* = 6488	Unadjusted Odds			Adjusted Odds		
	OR	95% CI	Model 1: aOR	95% CI	Model 2: aOR	95% CI
**Place of Residence**						
Rural (ref)	1.00	1.00	1.00	1.00	1.00	1.00
Urban	1.90	1.09–3.30	0.91	0.54–1.54	0.95	0.57–1.56
**Year**						
2016 (ref)	1.00	1.00	1.00	1.00	1.00	1.00
2017	1.08	0.68–1.72	1.09	0.66–1.79	1.64	0.97–2.78
2018	0.93	0.60–1.46	0.95	0.60–1.51	1.30	0.81–2.08
**Age (y)**						
15–24 (ref)	1.00	1.00	1.00	1.00	1.00	1.00
25–34	9.96	4.19–23.71	5.17	1.80–14.84	2.56	0.86–7.58
35–49	17.28	7.54–39.61	7.76	2.86–21.06	2.54	0.85–7.55
**Education**						
None (ref)	1.00	1.00	1.00	1.00	1.00	1.00
Primary	0.70	0.33–1.49	0.59	0.26–1.34	0.49	0.20–1.17
Secondary	1.01	0.53–1.93	1.01	0.48–2.14	0.97	0.44–2.13
Higher	2.05	1.01–4.17	1.31	0.59–2.90	1.39	0.58–3.32
**Marital Status**						
Married/living together (ref)	1.00	1.00	1.00	1.00	1.00	1.00
Divorced/separated	0.87	0.39–1.99	<0.001	<0.001–0.001	<0.001	<0.001–0.001
Never married	0.10	0.02–0.53	8.05	0.91–71.55	14.60	1.94–109.74
**Wealth Quintile**						
1 (lowest, ref)	1.00	1.00	1.00	1.00	1.00	1.00
2	0.68	0.22–2.15	0.64	0.21–1.94	0.53	0.16–1.77
3	0.93	0.31–2.81	1.43	0.51–4.08	1.12	0.40–3.16
4	2.56	0.84–6.59	3.37	1.11–10.26	2.68	0.94–7.61
5	3.74	1.37–10.23	4.50	1.39–14.57	3.68	1.18–11.45
**Family type**						
Monogamous (ref)	1.00	1.00	1.00	1.00	1.00	1.00
Polygamous	0.57	0.35–0.95	0.65	0.38–1.11	0.50	0.29–0.87
**State**						
Kaduna	1.00	1.00	1.00	1.00	1.00	1.00
Lagos	2.77	1.53–5.00	1.02	0.51–2.04	1.64	0.77–3.52
Taraba	0.64	0.12–3.33	1.31	0.27–6.40	2.38	0.49–11.45
Kano	3.54	1.50–8.37	2.05	0.97–4.34	2.47	0.92–6.59
River	0.76	0.37–1.57	0.31	0.14–0.66	0.51	0.21–1.24
Nasarawa	1.50	0.64–3.49	1.70	0.73–4.00	1.86	0.77–4.50
Anambra	1.62	0.86–3.05	0.74	0.37–1.47	1.85	0.84–4.10
**Final FP decider at facility**						
Respondent alone (ref)	1.00	1.00			1.00	1.00
Provider	2.48	1.39–4.42			2.29	1.25–4.22
Partner	0.13	0.05–0.38			0.13	0.04–0.44
Respondent and provider	1.74	0.94–3.23			1.55	0.85–2.85
Respondent and partner	0.76	0.51–1.12			0.58	0.37–0.89
Other	0.47	0.06–3.64			0.85	0.12–6.27
**Number of births**						
0–1 (ref)	1.00	1.00			1.00	1.00
2–4	14.35	4.82–42.74			3.57	1.01–12.61
5+	12.28	3.92–38.54			3.61	0.92–14.13
**More children desired**						
No (ref)	1.00	1.00			1.00	1.00
Yes	0.23	0.15–0.36			0.48	0.30–0.77
Cannot get pregnant	<0.001	<0.001–0.001			<0.001	<0.001–0.001
Undecided/Do not know	0.67	0.39–1.16			1.04	0.60–1.79
**Heard of LARCs**						
No (ref)	1.00	1.00			1.00	1.00
Yes	28.51	6.79–119.67			10.37	2.29–46.90

Note: OR = odds ratio; CI = confidence interval; LARCs = long-acting reversible contraceptives. Results are weighted to reflect the Nigeria female population. The first column shows the unadjusted odds ratio. In the adjusted models, Model 1: sociodemographic characteristics (maternal age, marital status, family type, place of residence, geopolitical zone, survey year wealth quintile, level of education); Model 2 added reproductive characteristics (number of births, intent for future children) and contraceptive characteristics (knowledge of LARCs, the contraceptive decision-maker at the facility).

**Table 5:** Weighted crude and adjusted odds of **implant** use among Nigerian women aged 15–49 using contraception.

*N* = 6488	Unadjusted Odds		Adjusted Odds			
	OR	95% CI	Model 1: aOR	95% CI	Model 2: aOR	95% CI
**Place of Residence**						
Rural (ref)	1.00	1.00	1.00	1.00	1.00	1.00
Urban	0.39	0.28–0.54	0.83	0.59–1.17	0.79	0.56–1.11
**Year**						
2016 (ref)	1.00	1.00	1.00	1.00	1.00	1.00
2017	1.02	0.79–1.32	1.05	0.79–1.40	1.36	1.01–1.83
2018	1.39	1.06–1.83	1.65	1.25–2.17	2.02	1.52–2.69
**Age (y)**						
15–24 (ref)	1.00	1.00	1.00	1.00	1.00	1.00
25–34	3.74	2.67–5.24	2.38	1.59–3.57	1.78	1.14–2.78
35–49	3.97	2.69–5.84	2.22	1.47–3.37	1.20	0.71–2.01
**Education**						
None (ref)	1.00	1.00	1.00	1.00	1.00	1.00
Primary	0.74	0.52–1.05	1.20	0.87–1.65	1.12	0.79–1.59
Secondary	0.40	0.27–0.58	1.35	0.96–1.90	1.40	0.96–2.05
Higher	0.35	0.24–0.51	1.44	0.95–2.18	1.53	0.98–2.39
**Marital Status**						
Married/living together (ref)	1.00	1.00	1.00	1.00	1.00	1.00
Divorced/separated	0.83	0.50–1.39	1.05	0.10–11.16	0.83	0.08–8.22
Never married	0.08	0.05–0.14	0.45	0.15–1.35	0.37	0.11–1.26
**Wealth Quintile**						
1 (lowest, ref)	1.00	1.00	1.00	1.00	1.00	1.00
2	1.01	0.63–1.64	1.38	0.97–1.96	1.43	0.99–2.06
3	0.62	0.35–1.10	1.65	1.07–2.55	1.67	1.04–2.68
4	0.39	0.23–0.64	1.19	0.76–1.85	1.17	0.72–1.91
5	0.41	0.25–0.66	1.57	0.94–2.61	1.64	0.94–2.87
**Family type**						
Monogamous (ref)	1.00	1.00	1.00	1.00	1.00	1.00
Polygamous	1.56	1.25–1.95	0.93	0.74–1.17	0.84	0.65–1.08
**State**						
Kaduna	1.00	1.00	1.00	1.00	1.00	1.00
Lagos	0.14	0.10–0.21	0.13	0.08–0.19	0.17	0.11–0.27
Taraba	0.20	0.10–0.40	0.28	0.15–0.52	0.38	0.21–0.68
Kano	0.45	0.31–0.65	0.38	0.26–0.56	0.41	0.26–0.63
River	0.15	0.10–0.24	0.15	0.09–0.23	0.25	0.15–0.40
Nasarawa	0.90	0.64–1.27	0.98	0.67–1.43	1.06	0.72–1.56
Anambra	0.11	0.07–0.19	0.10	0.06–0.18	0.17	0.10–0.30
**Final FP decider at facility**						
Respondent alone (ref)	1.00	1.00			1.00	1.00
Provider	2.13	1.02–4.47			0.94	0.50–1.77
Partner	0.39	0.23–0.66			0.49	0.28–0.84
Respondent and provider	2.47	1.73–3.54			1.31	0.91–1.90
Respondent and partner	0.92	0.69–1.21			0.75	0.56–1.00
Other	<0.001	<0.001–0.001			<0.001	<0.001–0.001
**Number of births**						
0–1 (ref)	1.00	1.00			1.00	1.00
2–4	4.33	3.04–6.17			1.56	0.97–2.52
5+	6.28	4.12–9.58			2.02	1.14–3.57
**More children desired**						
No (ref)	1.00	1.00			1.00	1.00
Yes	0.46	0.36–0.59			0.65	0.48–0.87
Cannot get pregnant	0.44	0.14–1.41			0.39	0.15–0.90
Undecided/do not know	0.76	0.53–1.10			0.74	0.50–1.09
**Heard of LARCs**						
No (ref)	1.00	1.00			1.00	1.00
Yes	18.31	7.87–42.57			7.95	3.40–18.60

Note: OR = odds ratio; CI = confidence interval; LARCs = long-acting reversible contraceptives. Results are weighted to reflect the Nigeria female population. The first column shows the unadjusted odds ratio. In the adjusted models, Model 1: sociodemographic characteristics (maternal age, marital status, family type, place of residence, geopolitical zone, survey year wealth quintile, level of education); Model 2 added reproductive characteristics (number of births, intent for future children) and contraceptive characteristics (knowledge of LARCs, the contraceptive decision-maker at the facility).

## Data Availability

The data used to generate the findings of this study are publicly available at: PMA https://www.pmadata.org/data/available-datasets (accessed on 15 September 2022).
